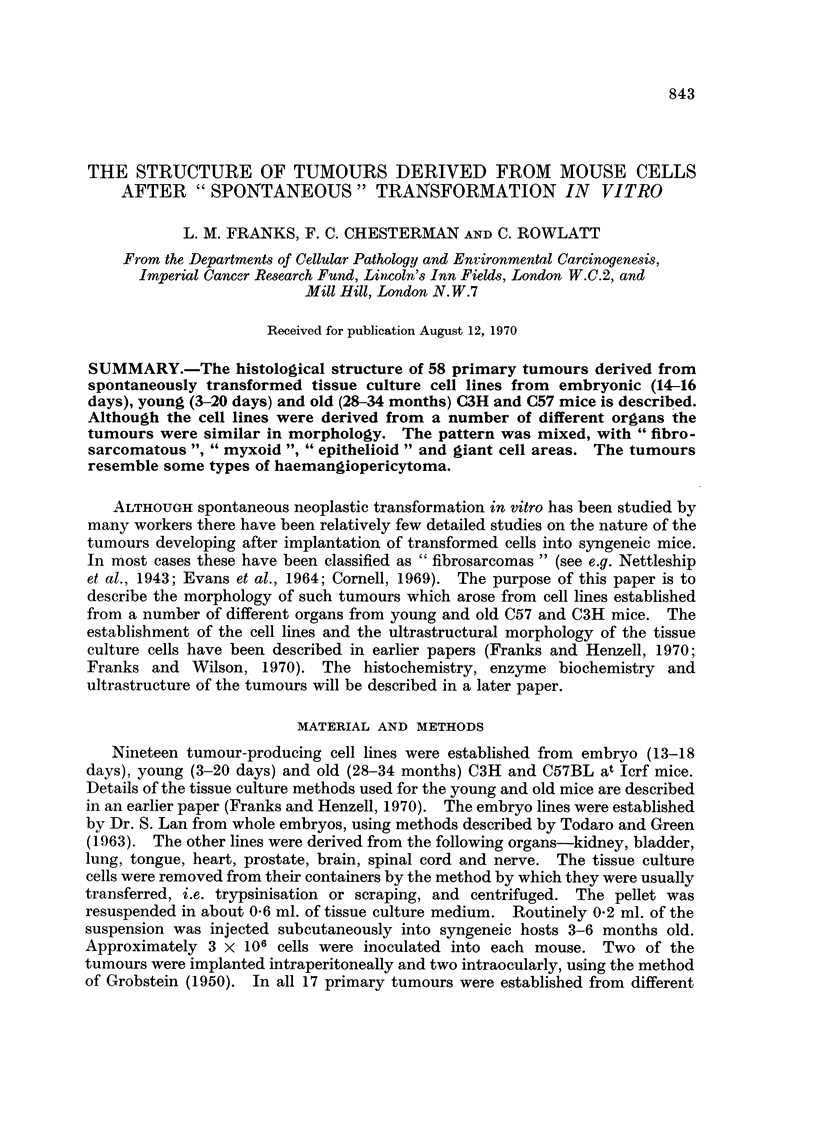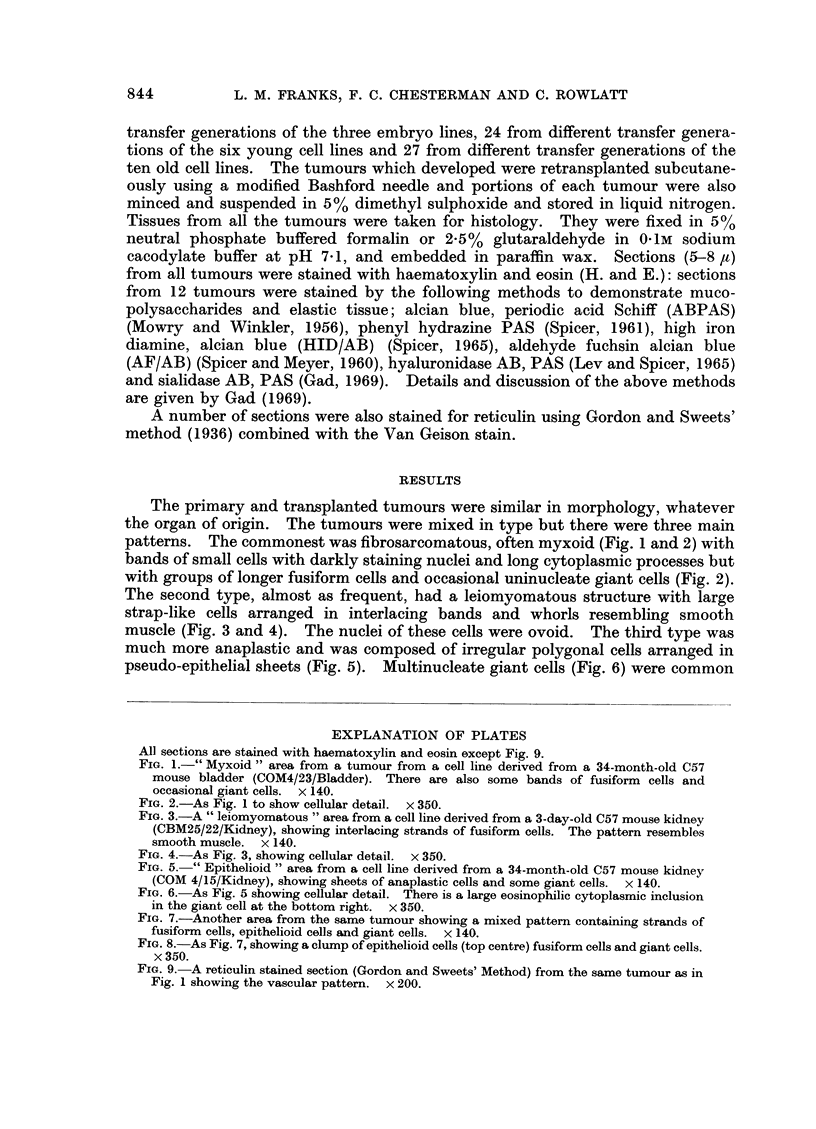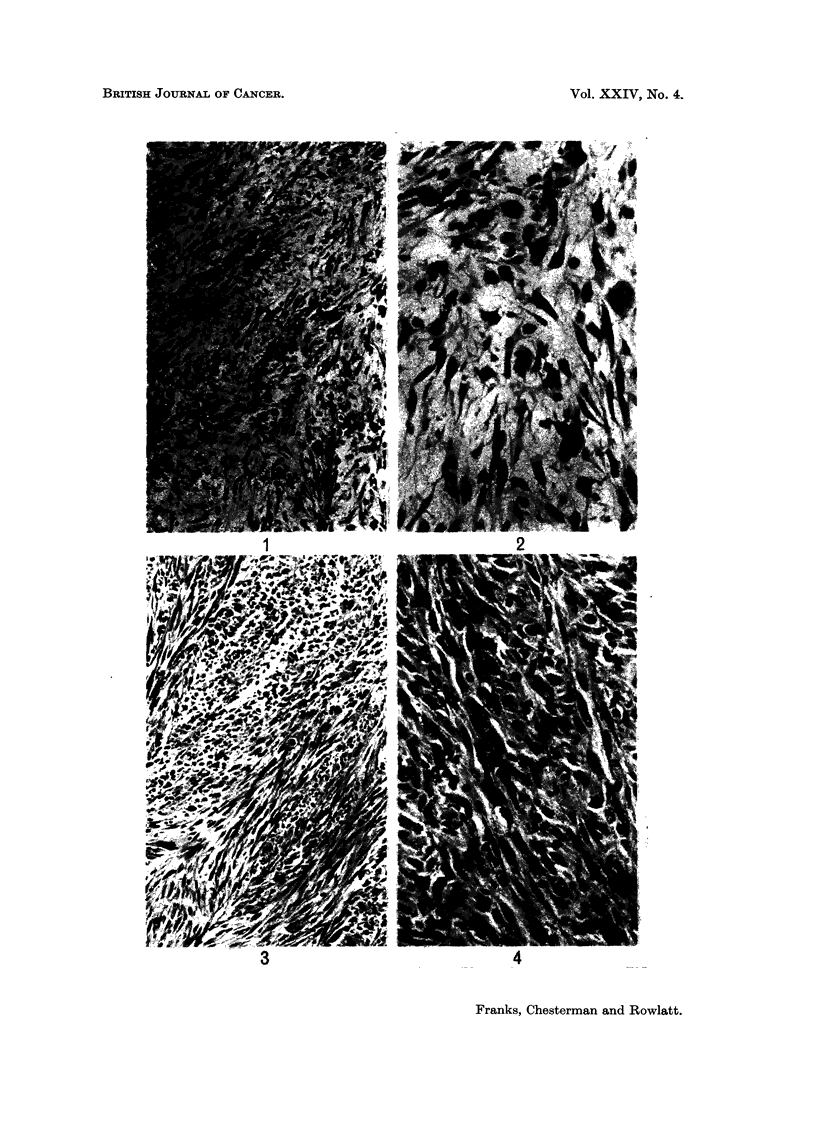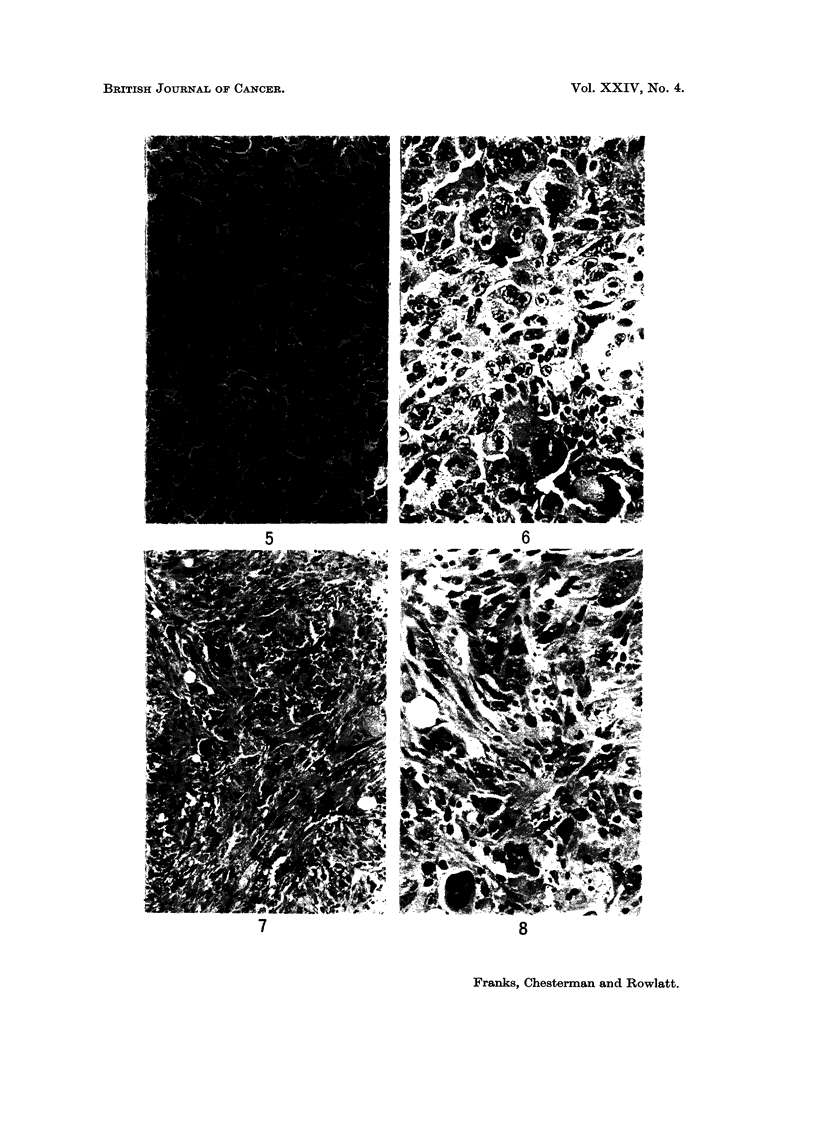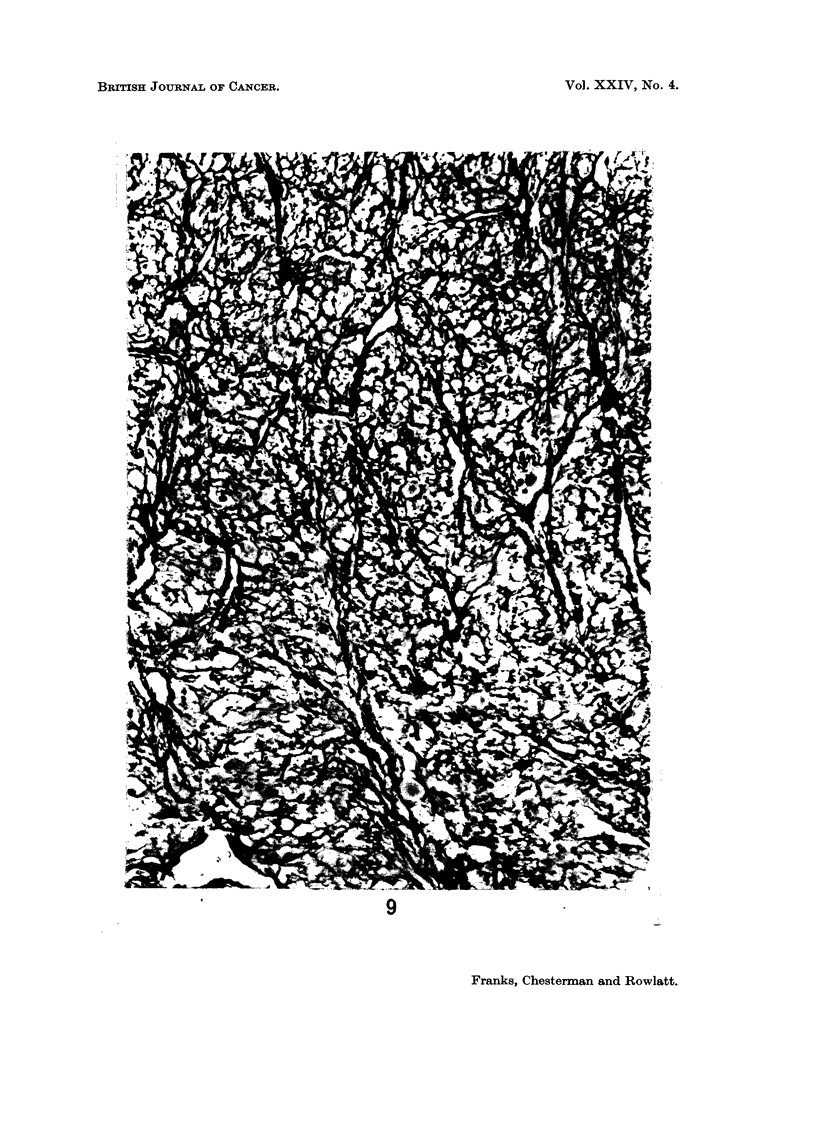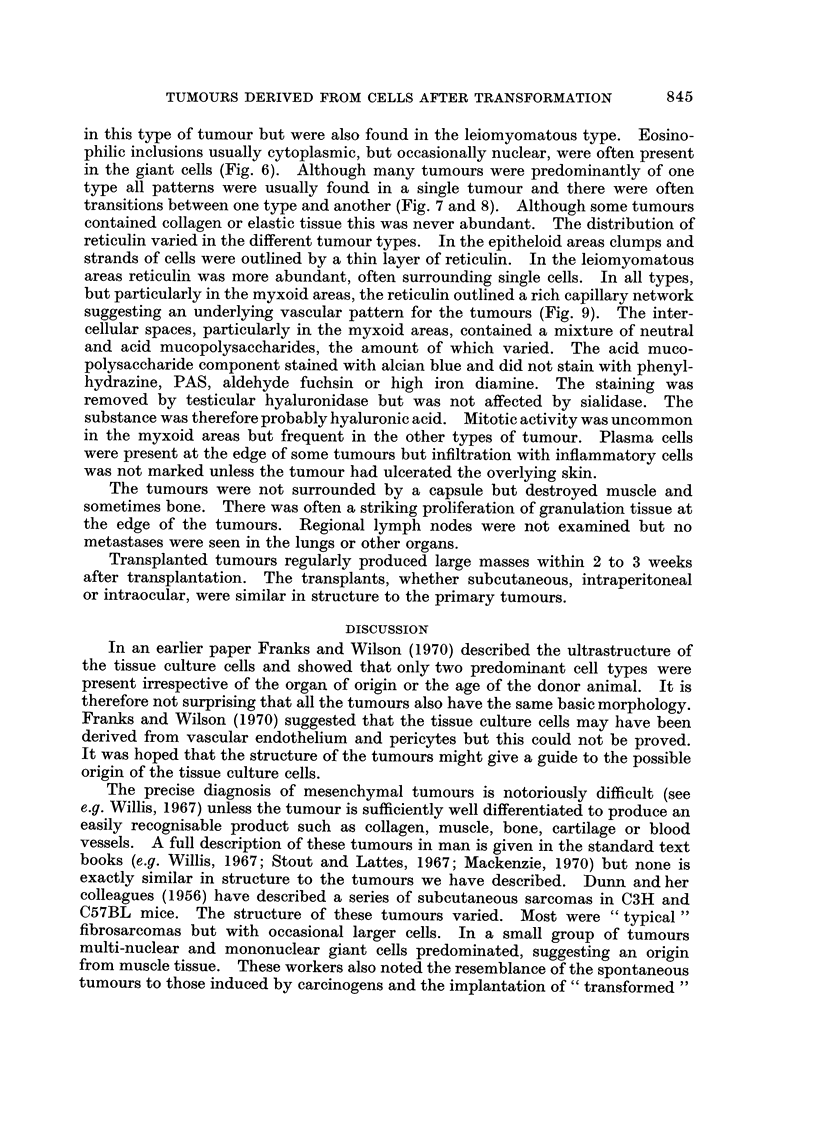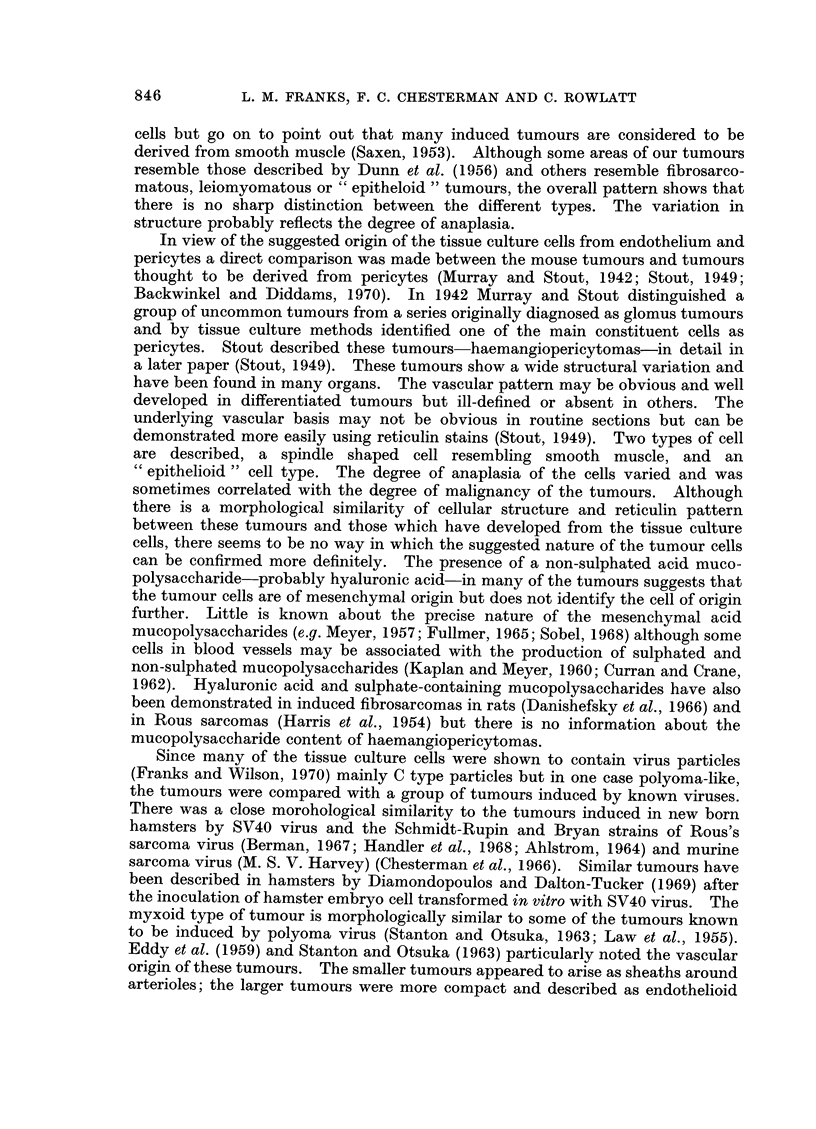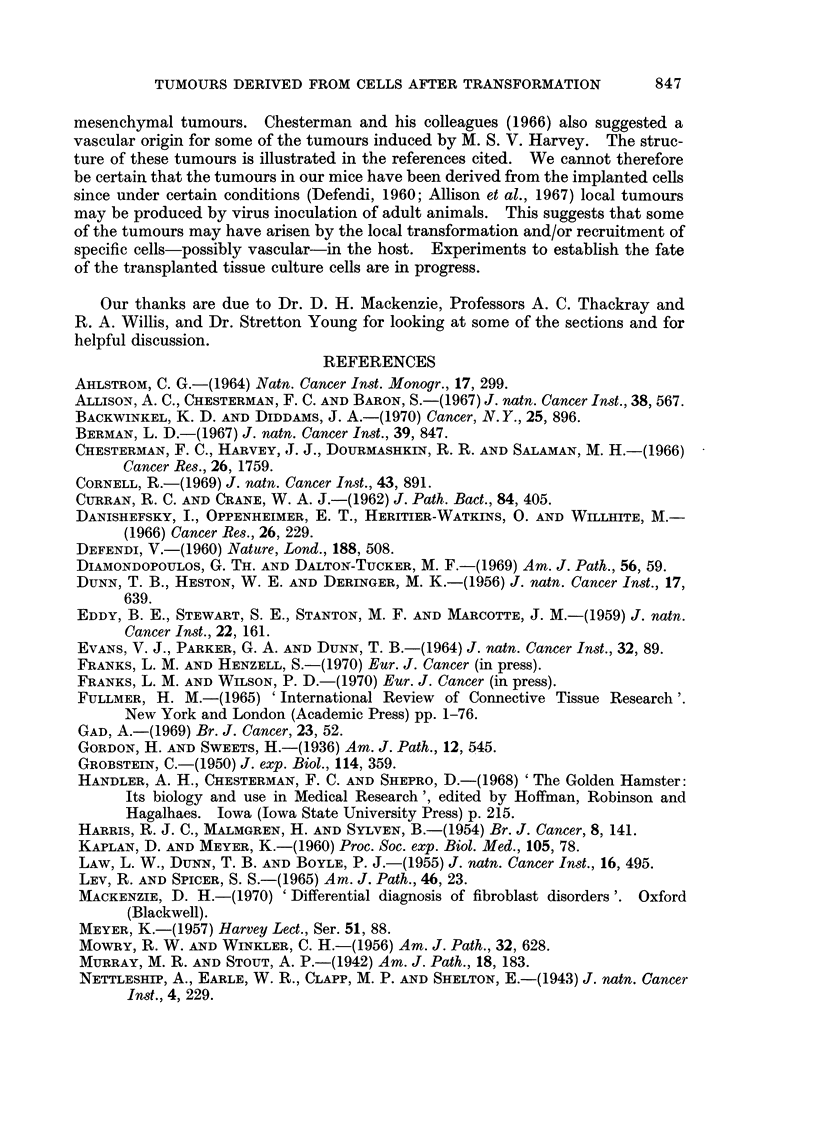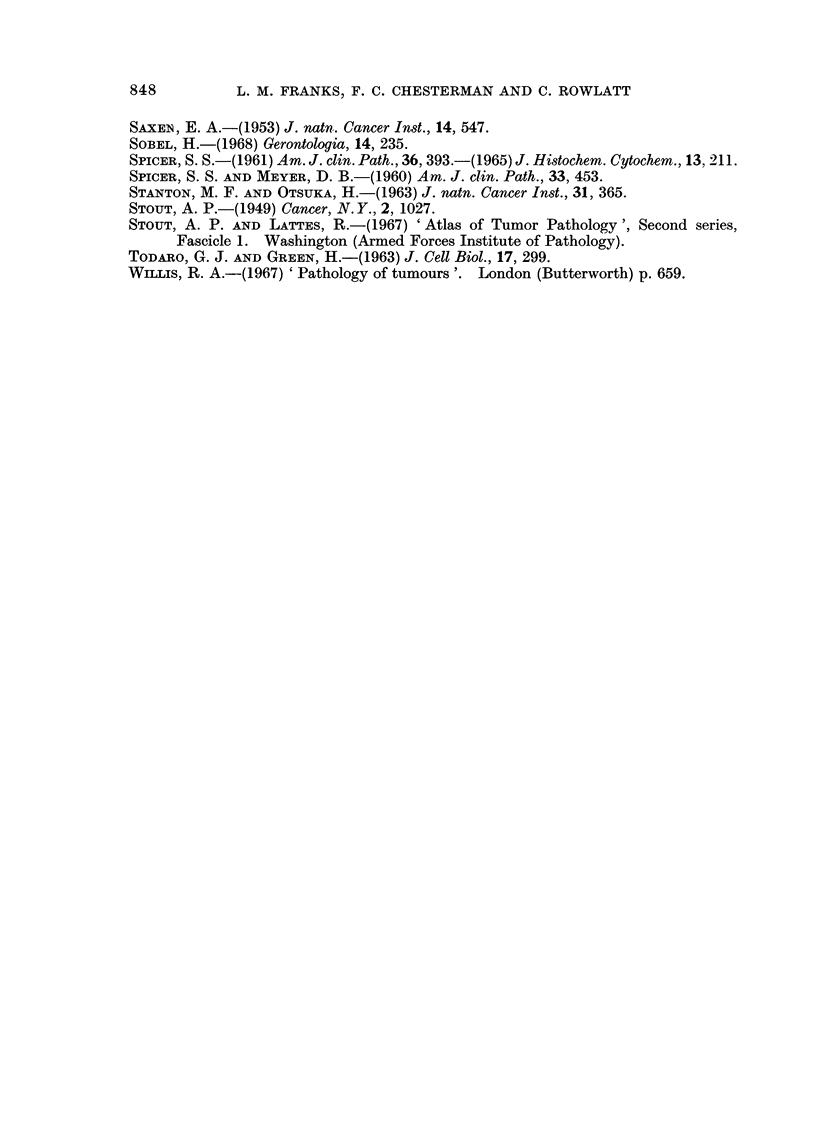# The Structure of Tumours Derived from Mouse Cells after “Spontaneous” Transformation in vitro

**DOI:** 10.1038/bjc.1970.99

**Published:** 1970-12

**Authors:** L. M. Franks, F. C. Chesterman, C. Rowlatt

## Abstract

**Images:**


### THE STRUCTURE OF TUMOURS DERIVED FROM MOUSE CELLS AFTER " SPONTANEOUS " TRANSFORMATION *IN VITRO*

L. M. FRANKS, F. C. CHESTERMAN AND C. ROWLATT

*From the Departments of Cellular Pathology and Environmental Carcinogenesis, Imperial Cancer Research Fund, Lincoln's Inn Fields, London W.C.2, and Mill Hill, London N.W.7*

Received for publication August 12, 1970

**SUMMARY.—The histological structure of 58 primary tumours derived from spontaneously transformed tissue culture cell lines from embryonic (14–16 days), young (3–20 days) and old (28–34 months) C3H and C57 mice is described. Although the cell lines were derived from a number of different organs the tumours were similar in morphology. The pattern was mixed, with " fibro-sarcomatous ", " myxoid ", " epithelioid " and giant cell areas. The tumours resemble some types of haemangiopericytoma.**

ALTHOUGH spontaneous neoplastic transformation *in vitro* has been studied by many workers there have been relatively few detailed studies on the nature of the tumours developing after implantation of transformed cells into syngeneic mice. In most cases these have been classified as " fibrosarcomas " (see *e.g.* Nettleship *et al.*, 1943; Evans *et al.*, 1964; Cornell, 1969). The purpose of this paper is to describe the morphology of such tumours which arose from cell lines established from a number of different organs from young and old C57 and C3H mice. The establishment of the cell lines and the ultrastructural morphology of the tissue culture cells have been described in earlier papers (Franks and Henzell, 1970; Franks and Wilson, 1970). The histochemistry, enzyme biochemistry and ultrastructure of the tumours will be described in a later paper.

### MATERIAL AND METHODS

Nineteen tumour-producing cell lines were established from embryo (13–18 days), young (3–20 days) and old (28–34 months) C3H and C57BL $a^t$ Icrf mice. Details of the tissue culture methods used for the young and old mice are described in an earlier paper (Franks and Henzell, 1970). The embryo lines were established by Dr. S. Lan from whole embryos, using methods described by Todaro and Green (1963). The other lines were derived from the following organs—kidney, bladder, lung, tongue, heart, prostate, brain, spinal cord and nerve. The tissue culture cells were removed from their containers by the method by which they were usually transferred, *i.e.* trypsinisation or scraping, and centrifuged. The pellet was resuspended in about 0·6 ml. of tissue culture medium. Routinely 0·2 ml. of the suspension was injected subcutaneously into syngeneic hosts 3–6 months old. Approximately $3 \times 10^6$ cells were inoculated into each mouse. Two of the tumours were implanted intraperitoneally and two intraocularly, using the method of Grobstein (1950). In all 17 primary tumours were established from different transfer generations of the three embryo lines, 24 from different transfer generations of the six young cell lines and 27 from different transfer generations of the ten old cell lines. The tumours which developed were retransplanted subcutaneously using a modified Bashford needle and portions of each tumour were also minced and suspended in 5% dimethyl sulphoxide and stored in liquid nitrogen. Tissues from all the tumours were taken for histology. They were fixed in 5% neutral phosphate buffered formalin or 2·5% glutaraldehyde in 0·1M sodium cacodylate buffer at pH 7·1, and embedded in paraffin wax. Sections (5–8 $\mu$) from all tumours were stained with haematoxylin and eosin (H. and E.): sections from 12 tumours were stained by the following methods to demonstrate mucopolysaccharides and elastic tissue; alcian blue, periodic acid Schiff (ABPAS) (Mowry and Winkler, 1956), phenyl hydrazine PAS (Spicer, 1961), high iron diamine, alcian blue (HID/AB) (Spicer, 1965), aldehyde fuchsin alcian blue (AF/AB) (Spicer and Meyer, 1960), hyaluronidase AB, PAS (Lev and Spicer, 1965) and sialidase AB, PAS (Gad, 1969). Details and discussion of the above methods are given by Gad (1969).

A number of sections were also stained for reticulin using Gordon and Sweets' method (1936) combined with the Van Geison stain.

### RESULTS

The primary and transplanted tumours were similar in morphology, whatever the organ of origin. The tumours were mixed in type but there were three main patterns. The commonest was fibrosarcomatous, often myxoid (Fig. 1 and 2) with bands of small cells with darkly staining nuclei and long cytoplasmic processes but with groups of longer fusiform cells and occasional uninucleate giant cells (Fig. 2). The second type, almost as frequent, had a leiomyomatous structure with large strap-like cells arranged in interlacing bands and whorls resembling smooth muscle (Fig. 3 and 4). The nuclei of these cells were ovoid. The third type was much more anaplastic and was composed of irregular polygonal cells arranged in pseudo-epithelial sheets (Fig. 5). Multinucleate giant cells (Fig. 6) were common

---

### EXPLANATION OF PLATES

All sections are stained with haematoxylin and eosin except Fig. 9.

FIG. 1.—" Myxoid " area from a tumour from a cell line derived from a 34-month-old C57 mouse bladder (COM4/23/Bladder). There are also some bands of fusiform cells and occasional giant cells. ×140.

FIG. 2.—As Fig. 1 to show cellular detail. ×350.

FIG. 3.—A " leiomyomatous " area from a cell line derived from a 3-day-old C57 mouse kidney (CBM25/22/Kidney), showing interlacing strands of fusiform cells. The pattern resembles smooth muscle. ×140.

FIG. 4.—As Fig. 3, showing cellular detail. ×350.

FIG. 5.—" Epithelioid " area from a cell line derived from a 34-month-old C57 mouse kidney (COM 4/15/Kidney), showing sheets of anaplastic cells and some giant cells. ×140.

FIG. 6.—As Fig. 5 showing cellular detail. There is a large eosinophilic cytoplasmic inclusion in the giant cell at the bottom right. ×350.

FIG. 7.—Another area from the same tumour showing a mixed pattern containing strands of fusiform cells, epithelioid cells and giant cells. ×140.

FIG. 8.—As Fig. 7, showing a clump of epithelioid cells (top centre) fusiform cells and giant cells. ×350.

FIG. 9.—A reticulin stained section (Gordon and Sweets' Method) from the same tumour as in Fig. 1 showing the vascular pattern. ×200.

Franks, Chesterman and Rowlatt.

5

6

7

8

Franks, Chesterman and Rowlatt.

9

Franks, Chesterman and Rowlatt.

in this type of tumour but were also found in the leiomyomatous type. Eosinophilic inclusions usually cytoplasmic, but occasionally nuclear, were often present in the giant cells (Fig. 6). Although many tumours were predominantly of one type all patterns were usually found in a single tumour and there were often transitions between one type and another (Fig. 7 and 8). Although some tumours contained collagen or elastic tissue this was never abundant. The distribution of reticulin varied in the different tumour types. In the epitheloid areas clumps and strands of cells were outlined by a thin layer of reticulin. In the leiomyomatous areas reticulin was more abundant, often surrounding single cells. In all types, but particularly in the myxoid areas, the reticulin outlined a rich capillary network suggesting an underlying vascular pattern for the tumours (Fig. 9). The intercellular spaces, particularly in the myxoid areas, contained a mixture of neutral and acid mucopolysaccharides, the amount of which varied. The acid mucopolysaccharide component stained with alcian blue and did not stain with phenylhydrazine, PAS, aldehyde fuchsin or high iron diamine. The staining was removed by testicular hyaluronidase but was not affected by sialidase. The substance was therefore probably hyaluronic acid. Mitotic activity was uncommon in the myxoid areas but frequent in the other types of tumour. Plasma cells were present at the edge of some tumours but infiltration with inflammatory cells was not marked unless the tumour had ulcerated the overlying skin.

The tumours were not surrounded by a capsule but destroyed muscle and sometimes bone. There was often a striking proliferation of granulation tissue at the edge of the tumours. Regional lymph nodes were not examined but no metastases were seen in the lungs or other organs.

Transplanted tumours regularly produced large masses within 2 to 3 weeks after transplantation. The transplants, whether subcutaneous, intraperitoneal or intraocular, were similar in structure to the primary tumours.

### DISCUSSION

In an earlier paper Franks and Wilson (1970) described the ultrastructure of the tissue culture cells and showed that only two predominant cell types were present irrespective of the organ of origin or the age of the donor animal. It is therefore not surprising that all the tumours also have the same basic morphology. Franks and Wilson (1970) suggested that the tissue culture cells may have been derived from vascular endothelium and pericytes but this could not be proved. It was hoped that the structure of the tumours might give a guide to the possible origin of the tissue culture cells.

The precise diagnosis of mesenchymal tumours is notoriously difficult (see *e.g.* Willis, 1967) unless the tumour is sufficiently well differentiated to produce an easily recognisable product such as collagen, muscle, bone, cartilage or blood vessels. A full description of these tumours in man is given in the standard text books (*e.g.* Willis, 1967; Stout and Lattes, 1967; Mackenzie, 1970) but none is exactly similar in structure to the tumours we have described. Dunn and her colleagues (1956) have described a series of subcutaneous sarcomas in C3H and C57BL mice. The structure of these tumours varied. Most were " typical " fibrosarcomas but with occasional larger cells. In a small group of tumours multi-nuclear and mononuclear giant cells predominated, suggesting an origin from muscle tissue. These workers also noted the resemblance of the spontaneous tumours to those induced by carcinogens and the implantation of " transformed "

cells but go on to point out that many induced tumours are considered to be derived from smooth muscle (Saxen, 1953). Although some areas of our tumours resemble those described by Dunn *et al.* (1956) and others resemble fibrosarcomatous, leiomyomatous or " epitheloid " tumours, the overall pattern shows that there is no sharp distinction between the different types. The variation in structure probably reflects the degree of anaplasia.

In view of the suggested origin of the tissue culture cells from endothelium and pericytes a direct comparison was made between the mouse tumours and tumours thought to be derived from pericytes (Murray and Stout, 1942; Stout, 1949; Backwinkel and Diddams, 1970). In 1942 Murray and Stout distinguished a group of uncommon tumours from a series originally diagnosed as glomus tumours and by tissue culture methods identified one of the main constituent cells as pericytes. Stout described these tumours—haemangiopericytomas—in detail in a later paper (Stout, 1949). These tumours show a wide structural variation and have been found in many organs. The vascular pattern may be obvious and well developed in differentiated tumours but ill-defined or absent in others. The underlying vascular basis may not be obvious in routine sections but can be demonstrated more easily using reticulin stains (Stout, 1949). Two types of cell are described, a spindle shaped cell resembling smooth muscle, and an " epithelioid " cell type. The degree of anaplasia of the cells varied and was sometimes correlated with the degree of malignancy of the tumours. Although there is a morphological similarity of cellular structure and reticulin pattern between these tumours and those which have developed from the tissue culture cells, there seems to be no way in which the suggested nature of the tumour cells can be confirmed more definitely. The presence of a non-sulphated acid mucopolysaccharide—probably hyaluronic acid—in many of the tumours suggests that the tumour cells are of mesenchymal origin but does not identify the cell of origin further. Little is known about the precise nature of the mesenchymal acid mucopolysaccharides (*e.g.* Meyer, 1957; Fullmer, 1965; Sobel, 1968) although some cells in blood vessels may be associated with the production of sulphated and non-sulphated mucopolysaccharides (Kaplan and Meyer, 1960; Curran and Crane, 1962). Hyaluronic acid and sulphate-containing mucopolysaccharides have also been demonstrated in induced fibrosarcomas in rats (Danishefsky *et al.*, 1966) and in Rous sarcomas (Harris *et al.*, 1954) but there is no information about the mucopolysaccharide content of haemangiopericytomas.

Since many of the tissue culture cells were shown to contain virus particles (Franks and Wilson, 1970) mainly C type particles but in one case polyoma-like, the tumours were compared with a group of tumours induced by known viruses. There was a close morohological similarity to the tumours induced in new born hamsters by SV40 virus and the Schmidt-Rupin and Bryan strains of Rous's sarcoma virus (Berman, 1967; Handler *et al.*, 1968; Ahlstrom, 1964) and murine sarcoma virus (M. S. V. Harvey) (Chesterman *et al.*, 1966). Similar tumours have been described in hamsters by Diamondopoulos and Dalton-Tucker (1969) after the inoculation of hamster embryo cell transformed *in vitro* with SV40 virus. The myxoid type of tumour is morphologically similar to some of the tumours known to be induced by polyoma virus (Stanton and Otsuka, 1963; Law *et al.*, 1955). Eddy *et al.* (1959) and Stanton and Otsuka (1963) particularly noted the vascular origin of these tumours. The smaller tumours appeared to arise as sheaths around arterioles; the larger tumours were more compact and described as endothelioid mesenchymal tumours. Chesterman and his colleagues (1966) also suggested a vascular origin for some of the tumours induced by M. S. V. Harvey. The structure of these tumours is illustrated in the references cited. We cannot therefore be certain that the tumours in our mice have been derived from the implanted cells since under certain conditions (Defendi, 1960; Allison *et al.*, 1967) local tumours may be produced by virus inoculation of adult animals. This suggests that some of the tumours may have arisen by the local transformation and/or recruitment of specific cells—possibly vascular—in the host. Experiments to establish the fate of the transplanted tissue culture cells are in progress.

Our thanks are due to Dr. D. H. Mackenzie, Professors A. C. Thackray and R. A. Willis, and Dr. Stretton Young for looking at some of the sections and for helpful discussion.

### REFERENCES

AHLSTROM, C. G.—(1964) *Natn. Cancer Inst. Monogr.*, **17**, 299.
ALLISON, A. C., CHESTERMAN, F. C. AND BARON, S.—(1967) *J. natn. Cancer Inst.*, **38**, 567.
BACKWINKEL, K. D. AND DIDDAMS, J. A.—(1970) *Cancer, N.Y.*, **25**, 896.
BERMAN, L. D.—(1967) *J. natn. Cancer Inst.*, **39**, 847.
CHESTERMAN, F. C., HARVEY, J. J., DOURMASHKIN, R. R. AND SALAMAN, M. H.—(1966) *Cancer Res.*, **26**, 1759.
CORNELL, R.—(1969) *J. natn. Cancer Inst.*, **43**, 891.
CURRAN, R. C. AND CRANE, W. A. J.—(1962) *J. Path. Bact.*, **84**, 405.
DANISHEFSKY, I., OPPENHEIMER, E. T., HERITIER-WATKINS, O. AND WILLHITE, M.—(1966) *Cancer Res.*, **26**, 229.
DEFENDI, V.—(1960) *Nature, Lond.*, **188**, 508.
DIAMONDOPOULOS, G. TH. AND DALTON-TUCKER, M. F.—(1969) *Am. J. Path.*, **56**, 59.
DUNN, T. B., HESTON, W. E. AND DERINGER, M. K.—(1956) *J. natn. Cancer Inst.*, **17**, 639.
EDDY, B. E., STEWART, S. E., STANTON, M. F. AND MARCOTTE, J. M.—(1959) *J. natn. Cancer Inst.*, **22**, 161.
EVANS, V. J., PARKER, G. A. AND DUNN, T. B.—(1964) *J. natn. Cancer Inst.*, **32**, 89.
FRANKS, L. M. AND HENZELL, S.—(1970) *Eur. J. Cancer* (in press).
FRANKS, L. M. AND WILSON, P. D.—(1970) *Eur. J. Cancer* (in press).
FULLMER, H. M.—(1965) ' International Review of Connective Tissue Research '. New York and London (Academic Press) pp. 1–76.
GAD, A.—(1969) *Br. J. Cancer*, **23**, 52.
GORDON, H. AND SWEETS, H.—(1936) *Am. J. Path.*, **12**, 545.
GROBSTEIN, C.—(1950) *J. exp. Biol.*, **114**, 359.
HANDLER, A. H., CHESTERMAN, F. C. AND SHEPRO, D.—(1968) ' The Golden Hamster: Its biology and use in Medical Research ', edited by Hoffman, Robinson and Hagalhaes. Iowa (Iowa State University Press) p. 215.
HARRIS, R. J. C., MALMGREN, H. AND SYLVEN, B.—(1954) *Br. J. Cancer*, **8**, 141.
KAPLAN, D. AND MEYER, K.—(1960) *Proc. Soc. exp. Biol. Med.*, **105**, 78.
LAW, L. W., DUNN, T. B. AND BOYLE, P. J.—(1955) *J. natn. Cancer Inst.*, **16**, 495.
LEV, R. AND SPICER, S. S.—(1965) *Am. J. Path.*, **46**, 23.
MACKENZIE, D. H.—(1970) ' Differential diagnosis of fibroblast disorders '. Oxford (Blackwell).
MEYER, K.—(1957) *Harvey Lect.*, Ser. **51**, 88.
MOWRY, R. W. AND WINKLER, C. H.—(1956) *Am. J. Path.*, **32**, 628.
MURRAY, M. R. AND STOUT, A. P.—(1942) *Am. J. Path.*, **18**, 183.
NETTLESHIP, A., EARLE, W. R., CLAPP, M. P. AND SHELTON, E.—(1943) *J. natn. Cancer Inst.*, **4**, 229.

SAXEN, E. A.—(1953) *J. natn. Cancer Inst.*, **14**, 547.
SOBEL, H.—(1968) *Gerontologia*, **14**, 235.
SPICER, S. S.—(1961) *Am. J. clin. Path.*, **36**, 393.—(1965) *J. Histochem. Cytochem.*, **13**, 211.
SPICER, S. S. AND MEYER, D. B.—(1960) *Am. J. clin. Path.*, **33**, 453.
STANTON, M. F. AND OTSUKA, H.—(1963) *J. natn. Cancer Inst.*, **31**, 365.
STOUT, A. P.—(1949) *Cancer, N.Y.*, **2**, 1027.
STOUT, A. P. AND LATTES, R.—(1967) 'Atlas of Tumor Pathology', Second series, Fascicle 1. Washington (Armed Forces Institute of Pathology).
TODARO, G. J. AND GREEN, H.—(1963) *J. Cell Biol.*, **17**, 299.
WILLIS, R. A.—(1967) 'Pathology of tumours'. London (Butterworth) p. 659.